# Berberine Extends Lifespan in *C. elegans* Through Multi-Target Synergistic Antioxidant Effects

**DOI:** 10.3390/antiox14040450

**Published:** 2025-04-09

**Authors:** Yingshuo Bei, Ting Wang, Shuwen Guan

**Affiliations:** School of Life Sciences, Jilin University, Changchun 130012, China

**Keywords:** anti-aging, berberine, *C. elegans*, antioxidant, insulin/IGF-1 signaling pathways

## Abstract

Aging is a process of gradual functional decline in complex physiological systems and is closely related to the occurrence of various diseases. Berberine, a bioactive alkaloid derived from *Coptis chinensis* (Huanglian), has emerged as a promising candidate for anti-aging interventions. This study comprehensively investigated the lifespan-extending effects and molecular mechanisms of berberine in *C. elegans* through integrated approaches including lifespan assays, locomotor activity analysis, oxidative stress challenges, and transcriptomic profiling. Furthermore, genetic models of mutant and transgenic worms were employed to delineate their interactions with the insulin/IGF-1 signaling (IIS) pathway. Our results demonstrate that berberine extended the mean lifespan of wild-type worms by 27%. By activating transcription factors such as DAF-16/FOXO, HSF-1, and SKN-1/NRF2, berberine upregulated antioxidant enzyme expression, reduced lipofuscin accumulation, and improved stress resistance. Transcriptomic analysis revealed significant changes in lipid metabolism-related genes, particularly in pathways involving fatty acid synthesis, degradation, and sphingolipid metabolism. These findings establish that berberine exerts multi-target anti-aging effects through coordinated activation of stress-responsive pathways and metabolic optimization, providing mechanistic insights for developing natural product-based geroprotective strategies.

## 1. Introduction

Aging represents a progressive decline in organismal homeostasis, characterized by multi-system physiological dysfunction at both cellular and systemic levels [1]. Elucidating its molecular mechanisms remains a central challenge in modern geroscience [2]. At the cellular level, this process manifests through mitochondrial dysfunction, proteostasis collapse, and compromised DNA repair mechanisms [3,4]. Systemically, it drives pathological manifestations including motor impairment, immunosenescence, and neurodegeneration [5]. Notably, age-associated diseases such as Alzheimer’s disease and type II diabetes now account for 70% of the global disease burden according to WHO statistics [6,7], underscoring the urgent need for translatable anti-aging interventions.

The disruption of reactive oxygen species (ROS) homeostasis has been identified as a critical molecular switch driving cellular senescence [8,9]. With advancing age, the gradual decline in metabolic function and natural reduction in synthesis/activity of antioxidant enzymes (e.g., superoxide dismutase, glutathione peroxidase) lead to ROS accumulation [10,11]. Excessive ROS triggers DNA damage and lipid peroxidation, thereby promoting cellular senescence. The activity of key oxidative stress transcription factors (FOXO/DAF-16, NRF2/SKN-1, HSF-1) is regulated by the IIS pathway [12,13]. Hyperactivation of the IIS pathway reduces antioxidant enzyme expression and weakens cellular defense against oxidative stress, accelerating senescence [14,15,16]. The IIS pathway has emerged as a prime therapeutic target for anti-aging interventions. Metformin, a classical IIS modulator, exerts anti-aging and metabolic regulatory effects through suppression of IIS overactivity [17,18,19]. However, its clinical application is limited by gastrointestinal side effects (e.g., diarrhea, nausea) and metabolic disorder risks (e.g., lactic acidosis) [20,21]. Rapamycin extends lifespan in various model organisms by indirectly inhibiting IIS downstream signaling [22], yet long-term use may induce metabolic abnormalities (hyperglycemia, hyperlipidemia) and immunosuppression [23,24]. These safety concerns have driven researchers to develop safer multi-target strategies, particularly exploring natural compounds that modulate aging-related pathways with favorable tolerability.

Berberine, a natural isoquinoline alkaloid extracted from medicinal plants (e.g., *Coptis chinensis*, *Phellodendron amurense*, *Berberis vulgaris*) [25,26], has demonstrated anti-aging potential in preclinical studies beyond its traditional antimicrobial applications [27]. Emerging evidence reveals its mechanisms involve AMPK-mediated metabolic regulation [28,29,30], autophagy modulation, and inflammasome suppression [31]. Emerging evidence confirms berberine’s lifespan-extending effects and neuroprotective properties against age-related pathologies [32], though these findings remain constrained by single-pathway analyses without systematic exploration of multi-target synergies or transcriptome-wide mechanistic validation.

**Figure 1:**
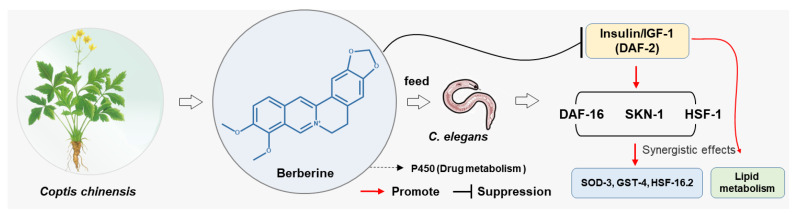
The bioactive component berberine from the traditional Chinese medicine *Coptis chinensis* exhibits multi-target synergistic anti-aging effects on *C. elegans*.

This study employs the *C. elegans* model to systematically investigate berberine’s anti-aging mechanisms (Figure 1). We demonstrate that berberine extends lifespan while enhancing motility and reducing lipofuscin accumulation-key markers of healthspan improvement. Mechanistically, it activates the DAF-16/SKN-1/HSF-1 antioxidant network downstream of IIS inhibition, upregulating SOD-3, HSP-16.2, and GST-4 expression. Transcriptomic analyses further reveal reshapes lipid metabolism pathways and regulate lysosomal function, suggesting multi-modal protection against cellular senescence. As a Chinese Pharmacopoeia-listed OTC drug with millennia of safe usage, berberine holds unique translational potential for developing precision anti-aging strategies and preventive therapies for age-related pathologies.

## 2. Materials and Methods

### 2.1. Preparation of Berberine Solution

Berberine has low solubility in water; therefore, dimethyl sulfoxide (DMSO) was chosen as a co-solvent to dissolve it. An appropriate amount of berberine was weighed and dissolved in DMSO to prepare a berberine stock solution containing 2% DMSO. This stock solution was then mixed with *E. coli* OP50 bacterial suspension to prepare working solutions of berberine containing 0.2% DMSO and 40, 80, and 160 µg/mL of berberine, respectively. For use, the prepared berberine working solution was spread onto the surface of the culture medium until it dried, forming a bacterial lawn.

### 2.2. C. elegans Strains

All strains were grown and maintained at 20 °C on nematode growth medium (NGM) agar plates seeded with *E. coli* OP50. The following *C. elegans* strains were used in this study: Wild-type N2 (*C. elegans* wild isolate, Bristol variety), TJ375 (*gpIs1 [hsp-16.2p::GFP]*), TJ356 (*zIs356 [daf-16p::daf-16a/b::GFP + rol-6(su1006)]*), CF1553 (*muIs84 [(pAD76) sod-3p::GFP + rol-6(su1006)]*), GR1307 (*daf-16(mgDf50)* I), PS3551 (*hsf-1(sy441)* I), and EU1 (*skn-1(zu67) IV/nT1 [unc-?(n754) let-?]* (IV; V)). The strain CB1370 (*daf-2(e1370)* III) was cultured at 16 °C, while the lifespan assays were conducted at 20 °C. Strains were provided by the Caenorhabditis Genetics Center (University of Minnesota, Minneapolis, MN, USA), which is funded by the NIH Office of Research Infrastructure Programs (P40OD011440).

### 2.3. Synchronization Methods

To obtain synchronized *C. elegans*, hermaphroditic worms in the egg-laying stage were selected and placed on an OP50-seeded culture medium for 4 h to lay eggs [33]. They were allowed to lay eggs on the culture medium for 4 h. Subsequently, the egg-laying *C. elegans* were transferred to another culture medium, and the eggs were retained. The *C. elegans* that hatched from these eggs in the culture medium were considered synchronized. For large-scale acquisition of worms, the NGM culture medium containing *C. elegans* in the peak egg-laying period was rinsed with M9 buffer to collect the worms into a centrifuge tube. The sample was allowed to stand for 1 min, the supernatant was discarded, and the worms were resuspended in an M9 buffer. This process was repeated three times. Then, 1 mL of lysis buffer was added, vortexed for 2 min, and centrifuged at low speed to remove the supernatant. The sample was washed once with M9 buffer, resuspended, and transferred onto an NGM culture medium seeded with *E. coli* OP50. The eggs were allowed to hatch to obtain synchronized *C. elegans*.

### 2.4. Lifespan Assay

A blank control group and berberine concentration gradient groups (40, 80, and 160 μg/mL) were set up. At least 70 synchronized L4-stage *C. elegans* were placed on NGM agar plates seeded with an adequate amount of *E. coli* OP50 and cultured at 20 °C. Starting from the day the worms were transferred to the plates, the survival status was recorded every 24 h by counting the number of dead worms. Worms that died accidentally (e.g., due to desiccation or crawling off the plate) were excluded from the mortality count. After each count, the surviving worms were transferred to fresh NGM plates. The day of synchronization was designated as Day 0 of the lifespan experiment. Death was determined by the absence of any response when the head of the worm was gently touched with a platinum wire. To prevent reproduction, 50 μM 5-fluorodeoxyuridine (FUdR) was added to the NGM plates throughout the experiment.

### 2.5. Bacterial Growth Assay

A total of 150 μL per well of a mixture containing LB medium, *E. coli* OP50, and berberine solution was added. The absorbance at 600 nm was measured at 0 h, then the plate was incubated at 37 °C, and measurements were taken every 2 h for 12 h to plot the bacterial growth curve [34].

### 2.6. Growth and Development

Adults in the egg-laying stage were transferred to NGM agar plates coated with a mixture of *E. coli* OP50 and berberine at a final concentration of 80 μg/mL. After 4 h, the adults were removed, leaving the eggs behind. A control group without berberine was also set up. This moment was recorded as 0 h. The growth of *C. elegans* was observed at 24 h, 48 h, and 96 h, with photographs taken for documentation. The body length and width of the worms were measured using ImageJ v1.54 software.

### 2.7. Locomotor Ability

At least 70 synchronized L4-stage worms were transferred to NGM agar plates coated with a mixture of *E. coli* OP50 and berberine at a final concentration of 80 μg/mL. The body bending frequency of *C. elegans* was measured on Day 3, Day 5, and Day 7 of cultivation to assess their locomotor ability [33]. The measurement method is as follows: A single *C. elegans* was picked into a 10 μL drop of M9 buffer. The worm would perform swimming movements within the droplet, characterized by body bending. A 30-sec video was recorded using a microscope-mounted camera to count the number of body bends, which served as an indicator of locomotor ability.

### 2.8. Pharyngeal Pumping Rate

At least 70 synchronized L4-stage worms were transferred to NGM agar plates coated with a mixture of *E. coli* OP50 and berberine at a final concentration of 80 μg/mL. On Day 5 of cultivation, the pharyngeal pumping rate of *C. elegans* was measured [14]. A microscope-mounted camera was used to observe the pharyngeal pumping movements of individual worms, and the number of pharyngeal contractions was recorded over 10 s.

### 2.9. Lipofuscin Content

At least 70 synchronized L4-stage worms were transferred to NGM agar plates coated with a mixture of *E. coli* OP50 and berberine at a final concentration of 80 μg/mL. On Day 12 of cultivation, the lipofuscin content was measured. The worms were anesthetized using 10 mM levamisole. The anesthetized worms were then placed on a 2% agar pad, which was cut and mounted onto a glass slide. The spontaneous fluorescence of lipofuscin in the *C. elegans* intestine was excited under ultraviolet light using a fluorescence microscope, and images were captured for documentation [35]. The fluorescence images were quantitatively analyzed using ImageJ v1.54 software.

### 2.10. Fertility Assay

Synchronized L4-stage worms were transferred to NGM agar plates coated with a mixture of *E. coli* OP50 and berberine at a final concentration of 80 μg/mL. Five worms were placed on each NGM plate to avoid overcrowding, which could affect fertility. Every 24 h, the worms were transferred to fresh NGM plates until the end of their egg-laying period. The NGM plates with eggs were then incubated at 20 °C for 48 h, and the number of hatched progeny was counted [33]. The average number of progeny per group was calculated to assess the impact of berberine on the fertility of *C. elegans*.

### 2.11. Heat Stress Assay

Heat stress and recovery culture were conducted to evaluate the thermotolerance of *C. elegans* under berberine treatment, which is applicable for studying the effects of drugs on the stress resistance of worms. Synchronized L4-stage *C. elegans* were transferred to NGM plates coated with a mixture of *E. coli* OP50 and berberine at a final concentration of 80 μg/mL, with 30 worms per group [34]. The plates were sealed with parafilm and placed horizontally in a 35 °C incubator for 5 h. After the treatment, the plates were removed, the parafilm was discarded, and the worms were transferred to a 20 °C incubator for a 12-h recovery period.

### 2.12. Acute Oxidative Stress Assay

Synchronized L4-stage *C. elegans* were transferred to NGM plates coated with *E. coli* OP50 and berberine at a final concentration of 80 μg/mL. A control group without berberine was also established, with 50 worms per group. After 5 days of cultivation, the worms were transferred to NGM plates containing 50 mM paraquat (PQ). Starting from the day of transfer, the survival of the worms was recorded every 24 h [14,36]. The remaining surviving worms were then transferred to freshly prepared paraquat plates, and the number of dead worms was recorded.

### 2.13. RNA Extraction, Reverse Transcription, and qRT-PCR

First, a large number of synchronized L4-stage *C. elegans* were obtained using the lysis method and treated with berberine for 5 days. After treatment, approximately 2000 worms from each group were collected into a centrifuge tube. The worms were washed three times with M9 buffer to remove residual bacterial solution on the surface, and excess buffer was removed as much as possible. The worms were then flash-frozen in liquid nitrogen and ground into powder using a handheld homogenizer on ice. After adding 500 μL of TransZol UP reagent and continuing to homogenize until the worms were fully lysed, the mixture was left to stand at room temperature for 5 min. RNA extraction was performed according to the instructions of the TransGen Biotech TransZol UP Plus RNA Kit (TransGen Biotech, Beijing, China). The extracted RNA was reverse-transcribed into cDNA using the TransGen Biotech TransScript One-step gDNA Removal and cDNA Synthesis SuperMix kit, following the manufacturer’s instructions. qRT-PCR was conducted using the TransGen Biotech TransScript Top Green qPCR SuperMix, with all steps strictly adhering to the kit’s instructions. The experimental data were processed using the 2^(−ΔΔCT)^ method to calculate the relative expression fold changes of the target genes. The expression level of the reference gene *act-1* was normalized to 1 as the standardization benchmark. The primer sequences are given in Table S3.

### 2.14. Whole-Genome Transcriptome

After five days of treatment, 2000 worms were collected from each group. The worms were washed and collected with M9 buffer that had been pre-chilled at 4 °C. The worms were allowed to settle. The supernatant was removed as much as possible. The samples were then rapidly frozen in liquid nitrogen. The samples were subsequently sent to Biomarker Technologies Corporation (Biomarker, Beijing, China) for transcriptome sequencing experiments.

### 2.15. Statistical Analysis

All experiments in this study included three or more biological replicates. Statistical analyses were performed using GraphPad Prism 8 software unless otherwise specified. Data are presented as mean ± standard deviation (mean ± SD). The mean lifespan was calculated from three independent lifespan experiments. The standard deviation is derived from these three replicates. Significance tests were conducted using the Student’s *t*-test (two-sided, unpaired). For all lifespan and paraquat survival curve experiments, survival curves were plotted using the Kaplan–Meier method. The Kaplan–Meier curve presents a set of representative data. Differences between control groups were compared using the log-rank test. In the statistical results, *p* < 0.05 is indicated by *, *p* < 0.01 by **, and *p* < 0.001 by ***.

## 3. Results

### 3.1. Effects of Berberine on the Lifespan and Biological Characteristics of C. elegans

**Figure 2:**
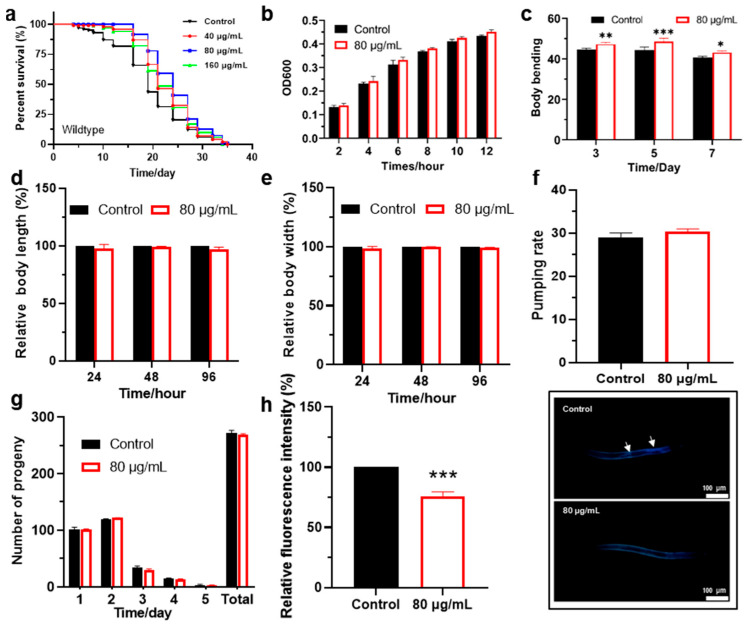
(**a**) Effects of different concentrations of berberine on the lifespan of *C. elegans*. (*n* > 70). (**b**) Impact of 80 μg/mL berberine on the growth of *E. coli* OP50. (**c**) Influence of berberine on the locomotor activity of *C. elegans*. (*n* > 70). The number of body bends within 30 s was measured in worms treated with berberine for 3, 5, and 7 days. (*n* = 60). (**d**) Effect of berberine on the body length of *C. elegans*. (*n* > 70). (**e**) Effect of berberine on the body width of *C. elegans*.(*n* > 70). (**f**) Impact of berberine on the pharyngeal pumping rate of wild-type *C. elegans*. (*n* > 70). (**g**) Effect of berberine on the reproductive capacity of wild-type *C. elegans*. (*n* = 5). (**h**) Berberine reduces the accumulation of lipofuscin in wild-type *C. elegans*. (*n* > 70). Right: Fluorescence images of lipofuscin in the worm body. Arrows indicate lipofuscin fluorescence in the intestine. * *p*  <  0.05, ** *p*  <  0.01 and *** *p*  <  0.001, two-tailed Student’s *t* test.

*C. elegans*, as a classic model organism, is widely used to study the mechanisms of aging and screen for anti-aging drugs due to its short lifespan and the conserved signaling pathways related to human aging [34,37]. In this study, *C. elegans* were treated with different concentrations of berberine (0, 40, 80, and 160 μg/mL), and it was found that berberine significantly extended the lifespan of wild-type worms in a dose-dependent manner (Figure 2a). Compared with the control group, the lifespan extension effect in the group treated with 80 µg/mL berberine was the most significant, reaching 27.3% (Table 1), which was significantly higher than the 19.6% observed in the 40 µg/mL group and the 14.7% in the 160 µg/mL group. Notably, the lifespan extension effect in the high-concentration group of 160 µg/mL was even lower than that in the 40 µg/mL group. Therefore, we selected the 80 µg/mL berberine concentration, which exhibited the optimal lifespan extension effect, for further experiments.

Berberine is known for its broad-spectrum antibacterial activity, capable of inhibiting the growth of various Gram-positive and Gram-negative bacteria [38,39]. However, the concentration of berberine used (80 μg/mL) did not significantly inhibit the growth of OP50 (Figure 2b). This may be because the concentration of berberine did not reach the minimum inhibitory concentration (MIC, >1 mg/mL) for *E. coli* [40]. Therefore, the impact of berberine on OP50 growth can be considered negligible under the experimental conditions. This result indicates that the lifespan-extending effect of berberine is not achieved by inhibiting food supply but rather by directly acting on the physiological processes of the worms.

Locomotor ability is an important indicator of the health status of worms, reflecting the normal function of their nervous system and muscles [41]. The locomotor ability of worms can be measured by indicators such as body bending frequency. Worms were treated with 80 μg/mL berberine for 3, 5, and 7 days, respectively, and then placed in droplets of M9 buffer to observe and record the number of body bends within 30 s at different treatment durations (Figure 2c). The results showed that the locomotor ability of worms treated with berberine was significantly enhanced, with the most significant effect observed after 5 days of treatment. Therefore, 5 days was chosen as the optimal treatment duration for subsequent experiments.

Growth, development, and reproduction are fundamental biological characteristics of *C. elegans* and are key processes for its survival and propagation [42]. As shown in Figure 2d,e, treatment with berberine had no significant effect on the body length and width of wild-type *C. elegans*, indicating that it does not have obvious adverse effects on the growth and development of the worms and is well-tolerated. The pharyngeal pumping rate of *C. elegans* treated with berberine did not significantly increase (*p* = 0.116), indicating that the lifespan-extending effect of berberine is not achieved by reducing food intake (Figure 2f). Furthermore, we assessed the impact of berberine on reproductive capacity by measuring the number of progeny produced by *C. elegans* after treatment. The results showed that berberine treatment did not reduce the number of offspring (Figure 2g), indicating that its lifespan-extending effects do not come at the expense of reproductive ability. In other words, the lifespan extension by berberine is not achieved by sacrificing reproductive capacity.

Lipofuscin is a product of cellular oxidative stress reactions, primarily composed of residues from the peroxidation of unsaturated fatty acids and protein oxidation, and serves as a biomarker of cellular aging and oxidative stress [43,44]. With increasing age, lipofuscin accumulates gradually in the intestinal tissues of *C. elegans*. Since lipofuscin emits blue fluorescence under ultraviolet light, its accumulation levels can be observed using a fluorescence microscope [45]. We found that berberine treatment significantly reduced the accumulation of lipofuscin in the intestines of adult *C. elegans* (Figure 2h). Specifically, in the control group, the intestinal fluorescence was pronounced, revealing the complete intestinal contour (indicated by arrows), demonstrating widespread accumulation of lipofuscin in the intestines. In contrast, the intestinal fluorescence in the berberine-treated group was significantly weaker, with only faint blue fluorescence observed on the body surface. This suggests that berberine may enhance the antioxidant capacity of cells or tissues, thereby reducing lipofuscin accumulation and slowing the aging process. The close association between lipofuscin accumulation and aging implies that its reduction may indicate enhanced antioxidant capacity in cells or tissues. This finding provides important evidence for exploring the mechanisms by which berberine extends lifespan.

In summary, berberine demonstrates significant anti-aging potential by extending the lifespan of *C. elegans*, enhancing their locomotor ability, and reducing lipofuscin accumulation. Its lifespan-extending effects are not dependent on dietary restriction mechanisms and do not significantly negatively impact the growth, development, or reproductive capacity of the worms, indicating its high safety profile. These findings provide important evidence for the development of berberine as an anti-aging drug and lay the foundation for further research into its antioxidant mechanisms.

### 3.2. Berberine Enhances Oxidative Stress Resistance in C. elegans

**Figure 3:**
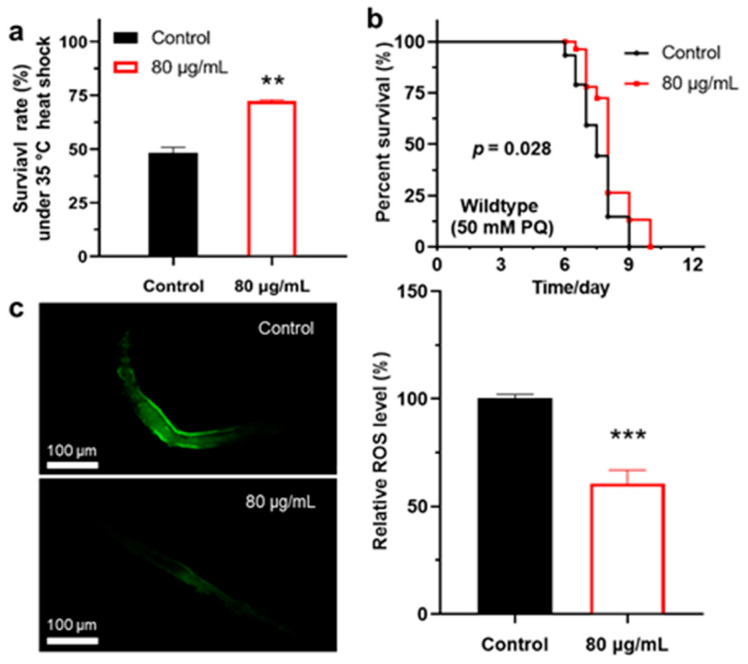
(**a**) Effects of berberine on the mortality rate of *C. elegans* after heat stress at 35 °C. (*n* = 30). (**b**) Impact of berberine on the lifespan of *C. elegans* under paraquat-induced oxidative stress. (*n* = 50). (**c**) Berberine reduces the accumulation of ROS in *C. elegans.* (*n* = 100). Left: Fluorescence images of ROS in the worm body. ** *p*  <  0.01 and *** *p*  <  0.001, two-tailed Student’s *t* test.

In the biology of aging, the disruption of ROS homeostasis is considered a key factor driving cellular senescence [46,47]. Numerous studies have revealed that enhancing antioxidant defenses not only significantly extends the lifespan of organisms but also improves their healthspan [48,49,50]. We found that berberine significantly reduces the accumulation of lipofuscin in the intestines of *C. elegans*, suggesting that it may delay aging by modulating antioxidant capacity. Based on these findings, we further explored whether berberine extends lifespan by enhancing the worms’ antioxidant capacity. To this end, we assessed the effects of berberine on stress resistance in *C. elegans* using two methods: heat stress and paraquat-induced oxidative stress. The results showed that berberine treatment significantly improved the survival of worms under heat stress, with an average survival rate increase of 24.1% (Figure 3a). Paraquat is a widely used herbicide, and its toxicity primarily stems from the generation of free radicals and the induction of cellular oxidative stress responses, which subsequently lead to oxidative damage in cells [51,52]. When worms are fed with berberine, their survival time in paraquat-exposed environments is significantly extended, demonstrating greater tolerance compared to the control group (Figure 3b). This suggests that berberine may alleviate paraquat-induced toxicity by modulating oxidative stress responses.

Additionally, we measured the levels of ROS in aging worms using the H2DCFDA fluorescent probe. The results indicated that ROS levels in worms treated with berberine were significantly reduced, with an average decrease of 39.4% (Figure 3c). This finding demonstrates that berberine possesses excellent antioxidant properties and may enhance the worms’ survival under stress conditions by modulating their antioxidant defense mechanisms, thereby contributing to lifespan extension.

### 3.3. Berberine Extends the Lifespan of C. elegans via the Insulin/IGF-1 Signaling Pathway

**Figure 4:**
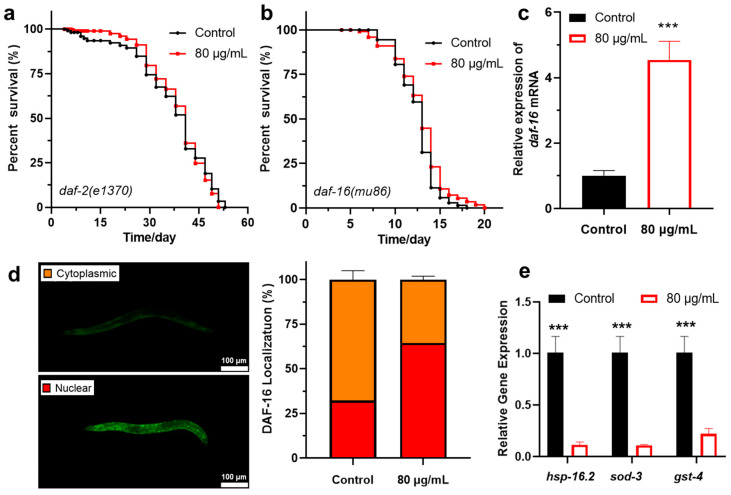
(**a**) The effect of berberine on the lifespan of *C. elegans daf-2(e1370)* mutant strain. (*n* > 70). (**b**) The effect of berberine on the lifespan of *C. elegans daf-16(maDf50)* mutant strain. (*n* > 70). (**c**) The effect of berberine on the expression level of *daf-16* mRNA in wild-type *C. elegans*. (**d**) Berberine promotes the nuclear translocation of the DAF-16 transcription factor. Right: DAF-16::GFP aggregates in the nucleus, showing green fluorescent spots. (*n* = 100). (**e**) The effect of berberine on the expression levels of downstream genes in *daf-16(maDf50)* mutant *C. elegans*. *** *p*  <  0.001, two-tailed Student’s *t* test.

To investigate whether berberine exerts its anti-aging effects through the IIS pathway, we conducted lifespan experiments using two mutant strains of *C. elegans*—*daf-2(e1370)* and *daf-16(maDf50)*. The results showed that berberine failed to extend the lifespan of these mutant strains (Figure 4a,b, Table 2). This indicates that the lifespan-extending effect of berberine is dependent on the DAF-2/DAF-16-mediated IIS signaling pathway. Additionally, we used qRT-PCR to examine the effect of berberine on *daf-16* mRNA expression. The results demonstrated that berberine significantly increased *daf-16* mRNA levels in wild-type *C. elegans* (Figure 4c). This finding further supports the notion that berberine extends worm lifespan by enhancing the regulation of the IIS pathway.

Specifically, DAF-16, a homolog of the FOXO family of transcription factors, plays a crucial role in regulating worm lifespan and stress responses [53,54,55]. When DAF-16/FOXO transcription factors are activated and translocated to the nucleus, they can regulate the expression of various antioxidant enzymes, such as SODs and GSH-Px [56]. Using the TJ356 transgenic *C. elegans* strain carrying the DAF-16::GFP fusion gene, we assessed the nuclear localization of DAF-16 before and after berberine treatment. The results showed that berberine significantly activated the translocation of DAF-16 to the nucleus, forming distinct fluorescent spots within the nucleus (Figure 4d), indicating substantial accumulation of DAF-16 in the nucleus.

Subsequently, we further examined the mRNA expression levels of *sod-3*, *gst-4*, and *hsp-16.2* in the *daf-16(maDf50)* mutant strain using qRT-PCR. The results showed that the absence of the *daf-16* gene led to a significant decrease in the expression of these downstream antioxidant enzymes (Figure 4e). This indicates that DAF-16 is essential for lifespan extension in *C. elegans*. However, these antioxidant enzymes still exhibited some level of expression, suggesting that berberine may also extend lifespan through other transcription factors or synergistic regulatory mechanisms.

**Figure 5:**
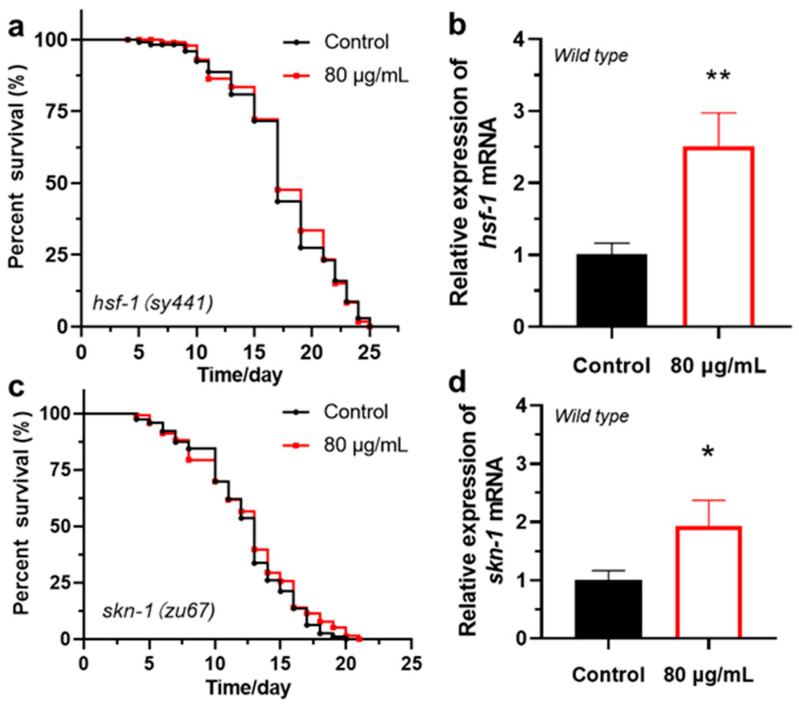
The lifespan-extending effect of berberine requires the participation of HSF-1 and SKN-1. (*n* > 70). (**a**) The effect of berberine on the lifespan of *C. elegans hsf-1(sy441)* mutant strain. (**b**) The effect of berberine on the expression level of *hsf-1* mRNA in wild-type *C. elegans*. (**c**) The effect of berberine on the lifespan of *C. elegans skn-1(zu67)* mutant strain. (*n* > 70). (**d**) The effect of berberine on the expression level of *skn-1* mRNA in wild-type *C. elegans*. * *p*  <  0.05, ** *p*  <  0.01, two-tailed Student’s *t* test.

In *C. elegans*, HSF-1 and SKN-1 downstream of the IIS pathway significantly enhance cellular antioxidant capacity and heat stress resistance by activating heat shock proteins (HSPs) and antioxidant enzyme genes [57,58]. To investigate whether berberine exerts its anti-aging effects through HSF-1 and SKN-1, we first conducted lifespan experiments using two mutant strains of *C. elegans*—*hsf-1*(sy441) and *skn-1*(zu67) (Figure 5a,c). The results showed that the lifespan of these two mutant strains was not extended under berberine feeding conditions (Table 3). This suggests that HSF-1 and SKN-1 may be key factors in berberine-mediated lifespan extension, playing indispensable roles in this process. Further qRT-PCR experiments indicated that berberine significantly increased the mRNA expression levels of *hsf-1* and *skn-1* in wild-type *C. elegans* (Figure 5b,d). These findings suggest that berberine may enhance the worms’ antioxidant capacity by upregulating the expression of HSF-1 and SKN-1, thereby activating downstream antioxidant genes.

**Figure 6:**
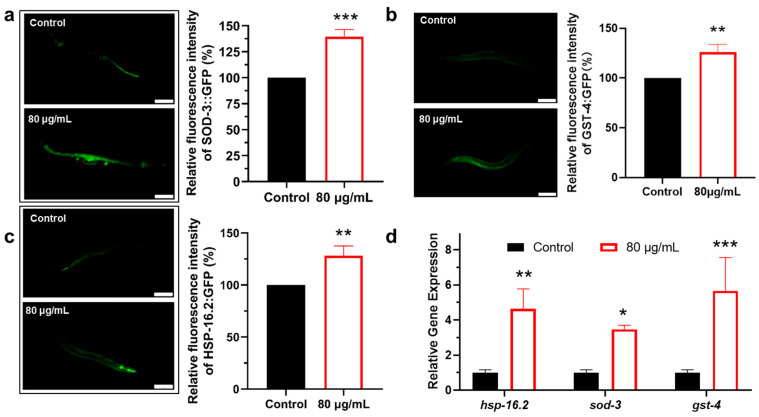
Effects of berberine on the expression levels of stress-responsive proteins in *C. elegans*. (**a**) The effect of berberine on the expression level of SOD-3::GFP in CF1553 transgenic *C. elegans*. Left: fluorescence images of SOD-3::GFP in the worm body. *n* = 100. (**b**) The effect of berberine on the expression level of GST-4::GFP in CL2166 transgenic *C. elegans*. Left: fluorescence images of GST-4::GFP in the worm body. *n* = 100. (**c**) The effect of berberine on the expression level of HSP-16.2::GFP in TJ375 transgenic *C. elegans*. *n* = 100. Relative fluorescence intensity of HSP-16.2::GFP. (**d**) The effect of berberine on the expression levels of stress-responsive genes in *C. elegans* measured by mRNA levels. Scale bar = 100 μm. * *p*  <  0.05, ** *p*  <  0.01 and *** *p*  <  0.001, two-tailed Student’s *t* test.

To evaluate whether berberine affects the expression of antioxidant stress genes downstream of HSF-1 and SKN-1 pathway, we utilized transgenic *C. elegans* carrying the SOD-3::GFP fusion. The results showed that in adult worms treated with berberine for 5 days, the expression level of SOD-3::GFP was significantly higher than that of the control group (Figure 6a). Similarly, in transgenic *C. elegans* carrying GST-4::GFP, the expression of GST-4::GFP was also significantly increased after berberine treatment (Figure 6b). These findings indicate that berberine significantly upregulates the expression of antioxidant stress genes in *C. elegans*.

Furthermore, during heat stress response in *C. elegans*, the survival capacity of worms is significantly enhanced, with HSF-1 playing a crucial role in regulating lifespan extension. As a key regulatory factor, HSF-1 enhances cellular stress tolerance and lifespan by activating heat shock proteins (such as HSP-16.2). Therefore, we used transgenic *C. elegans* carrying HSP-16.2::GFP to examine the effect of berberine on its expression. The results showed that the expression level of HSP-16.2::GFP was significantly upregulated in worms treated with berberine (Figure 6c).

Further, qRT-PCR experiments also confirmed that, as shown in Figure 6d, berberine significantly increased the mRNA expression levels of these genes. The lifespan extension mediated by berberine depends on its regulation of the antioxidant, and stress-related genes. The upregulation of the activity and expression levels of these genes is an essential mechanism by which berberine exerts its anti-aging effects.

### 3.4. Whole-Genome Transcriptional Analysis

**Figure 7:**
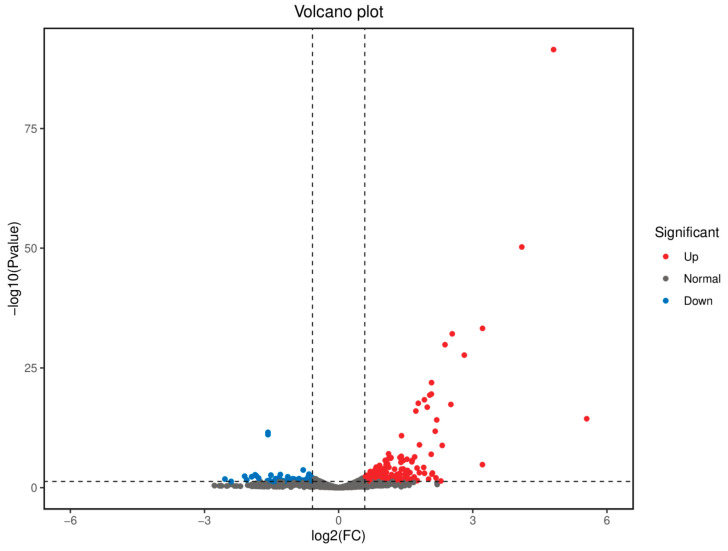
In *C. elegans* treated with berberine, there were 300 differentially expressed genes (FDR < 0.01, fold change > 2), among which 252 genes were significantly upregulated and 48 genes were significantly downregulated. (*n* > 2000).

To further elucidate the mechanisms by which berberine extends the lifespan of *C. elegans*, we conducted transcriptome sequencing (RNA-seq) analysis. In the experiment, after treating *C. elegans* with berberine for 5 days, the results showed significant differences in gene expression between berberine-treated and control worms. Through identification and analysis, we found that there were 300 differentially expressed genes (DEGs) in berberine-treated *C. elegans* compared to the control group (FDR < 0.01 and fold change > 2), including 252 significantly upregulated genes and 48 significantly downregulated genes (Figure 7). The identification of these DEGs provides an essential molecular basis for revealing the anti-aging mechanisms of berberine in *C. elegans*.

For the upregulated genes among the differentially expressed genes identified above, the Gene Ontology database (GO) was used for classification and description, which is divided into three categories: biological process, cellular component, and molecular function (Figure S1). The RNA-seq results showed that in the biological process category, upregulated genes were mainly enriched in innate immune response and oxidation–reduction process (Figure S2). In the cellular component category, upregulated genes were primarily enriched in the cytoskeleton and intracellular membrane-bounded organelle (Figure S3). In the molecular function category, upregulated genes were mainly enriched in heme binding, iron ion binding, and protein serine/threonine kinase activity (UDP-glycosyltransferase catalyzing glycosyl transfer reactions) (Figure S4). These various biological activities and signaling pathways are closely related to aging and cellular immune responses.

**Figure 8:**
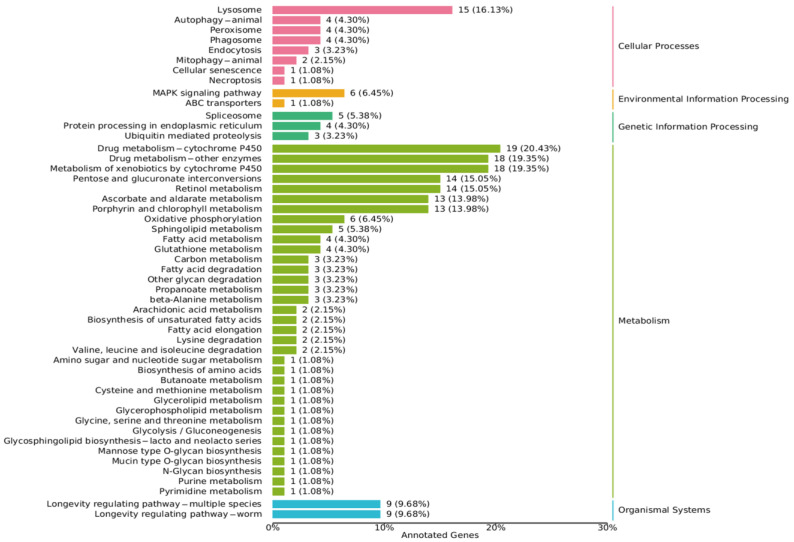
The KEGG pathway enrichment analysis of differentially expressed genes. The analysis reveals five major categories of enrichment: cellular processes, environmental information processing, genetic information processing, metabolism, and organismal systems.

Further analysis using KEGG pathway analysis revealed that in berberine-treated *C. elegans*, a large number of genes were enriched in the metabolism term, with drug metabolism-cytochrome P450 and drug metabolism-other enzymes accounting for 39.78% (37 annotated genes) of the enriched genes (Figure 8). In mammals, the metabolism of berberine is primarily accomplished by the cytochrome P450 enzyme system in the liver, especially subtypes such as *CYP3A4*, *CYP2D6*, and *CYP1A2* [59,60,61]. Analysis showed that after berberine treatment, significant changes occurred in the expression of genes related to drug metabolism in the worms, with upregulation of the cytochrome P450 gene family and the UDP-glucuronosyltransferase gene family. This suggests that these genes may be involved in the metabolic process of berberine in *C. elegans*.

**Figure 9:**
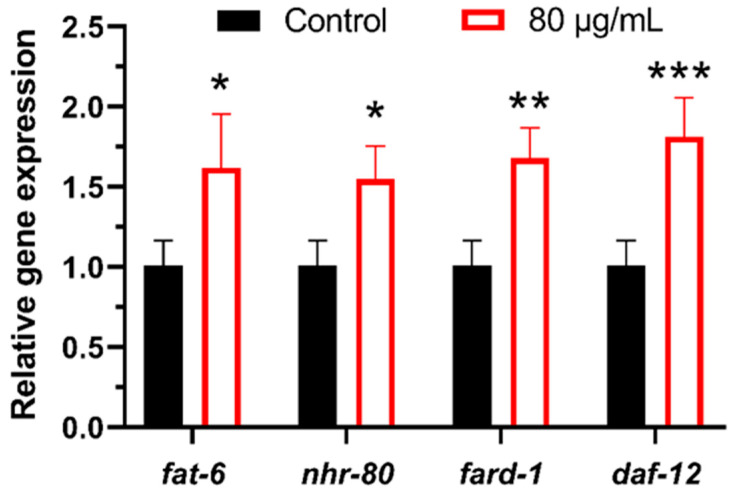
The effect of berberine on the expression levels of lipid metabolism-related genes in *C. elegans*. * *p*  <  0.05, ** *p*  <  0.01 and *** *p*  <  0.001, two-tailed Student’s *t* test.

Additionally, as shown in Figure 8, in lipid metabolism-related processes (including sphingolipid metabolism, fatty acid metabolism, fatty acid degradation, biosynthesis of unsaturated fatty acids, fatty acid elongation, glycerolipid metabolism, and glycerophospholipid metabolism), enrichment exceeded 19.37% (18 annotated genes). The IIS pathway in *C. elegans* regulates lipid metabolism by modulating multiple transcription factors and downstream genes. Among these, *daf-12* and *nhr-80* are key nuclear receptor transcription factors involved in regulating fat synthesis, storage, and breakdown [62,63,64]. *fard-1* and *fat-6* are downstream genes of the IIS pathway, encoding fatty acid synthase and fatty acid desaturase, respectively, and play important roles in lipid metabolism. The experimental results indicate that berberine significantly increased the expression levels of *daf-12*, *nhr-80*, *fard-1*, and *fat-6* (Figure 9). This regulatory mechanism may be related to the anti-aging and healthspan-extending effects of berberine, as optimized lipid metabolism can improve the worms’ energy balance and stress tolerance. These findings are corroborated by the transcriptomic data, further elucidating the mechanisms by which berberine regulates lipid metabolism.

In the cellular processes category, lysosome and autophagy were significantly enriched (Figure 8). One study found that berberine can activate the autophagy–lysosome pathway, reducing the accumulation of damaged proteins and organelles within cells. Reducing the accumulation of damaged proteins and organelles is a key mechanism for maintaining cellular health and extending lifespan [65,66]. Additionally, the MAPK signaling pathway was the most enriched pathway, primarily involved in stress response, immune response, and developmental processes [67,68,69]. In the organismal systems category, a large number of genes (9.68%, 9 annotated genes) were enriched in the longevity regulation pathway in berberine-treated *C. elegans*. Transcriptomics has revealed the multi-faceted molecular mechanisms by which berberine extends the lifespan of *C. elegans*. It acts through antioxidant defense, stress response, and metabolic regulation, activating complex metabolic pathways that optimize fatty acid metabolism, energy homeostasis, autophagy, and lysosomal function. These actions collectively enhance cellular health and significantly extend the lifespan of *C. elegans*.

We further compared the differentially expressed genes with the gene function annotations in the WormBase database and identified the top five most significantly upregulated and downregulated genes. These genes exhibited significant expression changes in *C. elegans* fed with berberine and are involved in various biological processes, including metabolic regulation, stress response, signal transduction, and cellular protection (Table S2). Among them, cytochrome P450 family genes (such as *cyp-35A1*, *cyp-35A5*, and *cyp-35B2*) play important roles in metabolizing xenobiotics, while other genes (such as *irg-6* and *ins-31*) may be involved in stress response and signal transduction (Table S3). By regulating the expression of these genes, berberine may enhance the worms’ metabolic capacity, stress tolerance, and cellular protection mechanisms, thereby extending their lifespan. These genetic changes are closely related to the anti-aging and metabolic regulatory effects of berberine, providing important clues for further research into its biological mechanisms.

## 4. Conclusions

This study demonstrates that berberine significantly enhances the antioxidant defense and stress resistance of *C. elegans* by activating key transcription factors (DAF-16/FOXO, HSF-1, and SKN-1) and upregulating the expression of antioxidant enzymes, thereby significantly extending its lifespan. This research highlights the therapeutic potential of berberine as a natural anti-aging agent. Its multi-target synergistic actions offer a promising avenue for developing precision anti-aging strategies based on natural products. Our findings not only advance the understanding of berberine’s mechanisms but also provide a foundation for exploring its clinical applications in delaying aging and preventing aging-associated diseases. However, it is also important to note that the anti-aging effects of berberine in *C. elegans* have several limitations. The findings from *C. elegans* may not directly apply to higher organisms, so more complex models (e.g., mammals) should be tested. Moreover, while we identified key transcriptional changes and antioxidant pathways, the full molecular mechanisms of berberine’s anti-aging effects are likely more complex and may require in-depth exploration through proteomics, metabolomics, and other omics approaches.

## Figures and Tables

**Table 1:** Effects of different concentrations of berberine on the mean lifespan of wild-type *C. elegans.* Data represent the mean lifespan derived from three independent lifespan experiments, with the mean and standard deviation calculated from these three replicates. *p* value compared to the control. ** *p*  <  0.01 and *** *p*  <  0.001.

Genotype	Treatment	Mean Lifespan (Mean ± SD, Day)	% (vs. Control)	*p* Value (Log-Rank Significance)
Wildtype	Control	19.2 ± 0.7	-	-
Wildtype	40 μg/mL	22.9 ± 0.6	19.6%	<0.01 **
Wildtype	80 μg/mL	24.4 ± 0.6	27.3%	<0.001 ***
Wildtype	160 μg/mL	22.0 ± 0.6	14.7%	<0.01 **

**Table 2:** Effects of berberine on the mean lifespan of *daf-2(e1370)* and *daf-16(mu86)* mutant *C. elegans.* Data represent the mean lifespan derived from three independent lifespan experiments, with the mean and standard deviation calculated from these three replicates. *p* value compared to the control.

Genotype	Treatment	Mean Lifespan (Mean ± SD, Day)	% (vs. Control)	*p* Value (Log-Rank Significance)
*daf-2(e1370)*	Control	36.0 ± 12.5	-	-
	80 μg/mL	38.3 ± 9.4	6.3%	0.97
*daf-16(mu86)*	Control	12.4 ± 2.1	-	-
	80 μg/mL	12.7 ± 2.8	2.3%	0.11

**Table 3:** Effects of berberine on the mean lifespan of hsf-1(sy441) and skn-1(zu67) mutant *C. elegans*. Data represent the mean lifespan derived from three independent lifespan experiments, with the mean and standard deviation calculated from these three replicates. *p* value compared to the control.

Genotype	Treatment	Mean Lifespan (Mean ± SD, Day)	% (vs. Control)	*p* Value (Log-Rank Significance)
*hsf-1(sy441)*	Control	17.2 ± 4.6	-	-
	80 μg/mL	17.4 ± 4.4	1.0%	0.87
*skn-1(zu67)*	Control	11.7 ± 3.8	-	-
	80 μg/mL	12.2 ± 4.1	1.8%	0.39

## Data Availability

The original contributions presented in this study are included in the article and Supplementary Materials. Further inquiries can be directed to the corresponding authors.

## Supplementary Materials

The following supporting information can be downloaded at https://www.mdpi.com/article/10.3390/antiox14040450/s1, Figure S1: Based on the Gene Ontology (GO term) database, the functions of genes are categorized and annotated; Figure S2: In the biological process, the upregulated genes are mainly enriched in innate immune response and oxidation–reduction process. Figure S3: In the cellular component, the upregulated genes are mainly enriched in cytoskeleton and intracellular membrane-bounded organelle. Figure S4: In the molecular function category, the upregulated genes are mainly enriched in heme binding, iron ion binding, and protein serine/threonine kinase activity. Table S1: The gene sequence for RT-qPCR; Table S2: The 5 genes with the most significant upregulation of gene expression by berberine in *C. elegans*; Table S3: The 5 genes with the most significant downregulation of gene expression by berberine in *C. elegans*.
