# Supplementary material for: Berberine Extends Lifespan in C. elegans Through Multi-Target Synergistic Antioxidant Effects
Source: Antioxidants (Basel). 2025 Apr 9;14(4):450. doi: 10.3390/antiox14040450 (PMC12024168; doi:10.3390/antiox14040450)
Supplement: Supplementary file 1 [file antioxidants-14-00450-s001.zip › antioxidants-3530559-supplementary.pdf]

Berberine Extends Lifespan in *C. elegans* Through Multi-Target Synergistic Antioxidant Effects

Yingshuo Bei, Ting Wang and Shuwen Guan *

School of Life Sciences, Jilin University, Changchun 130012, China
* Correspondence: guanshuwen@jlu.edu.cn; Tel.: +86-135-0432-8390

Figure S1-S4.
Table S1-S3.

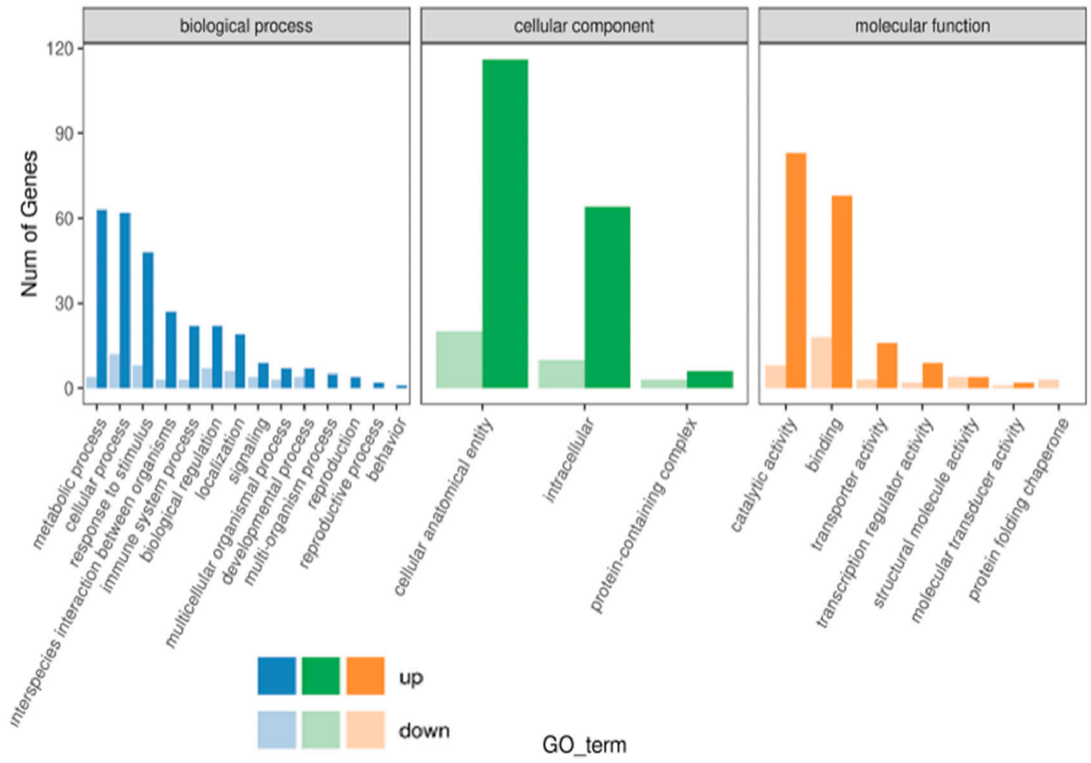

Figure S1. Based on the Gene Ontology (GO term) database, the functions of genes are categorized and annotated. The GO database classifies gene functions into three main aspects: Biological Process, Cellular Component, and Molecular Function, which are used to understand the specific roles of differentially expressed genes within cells and organisms.

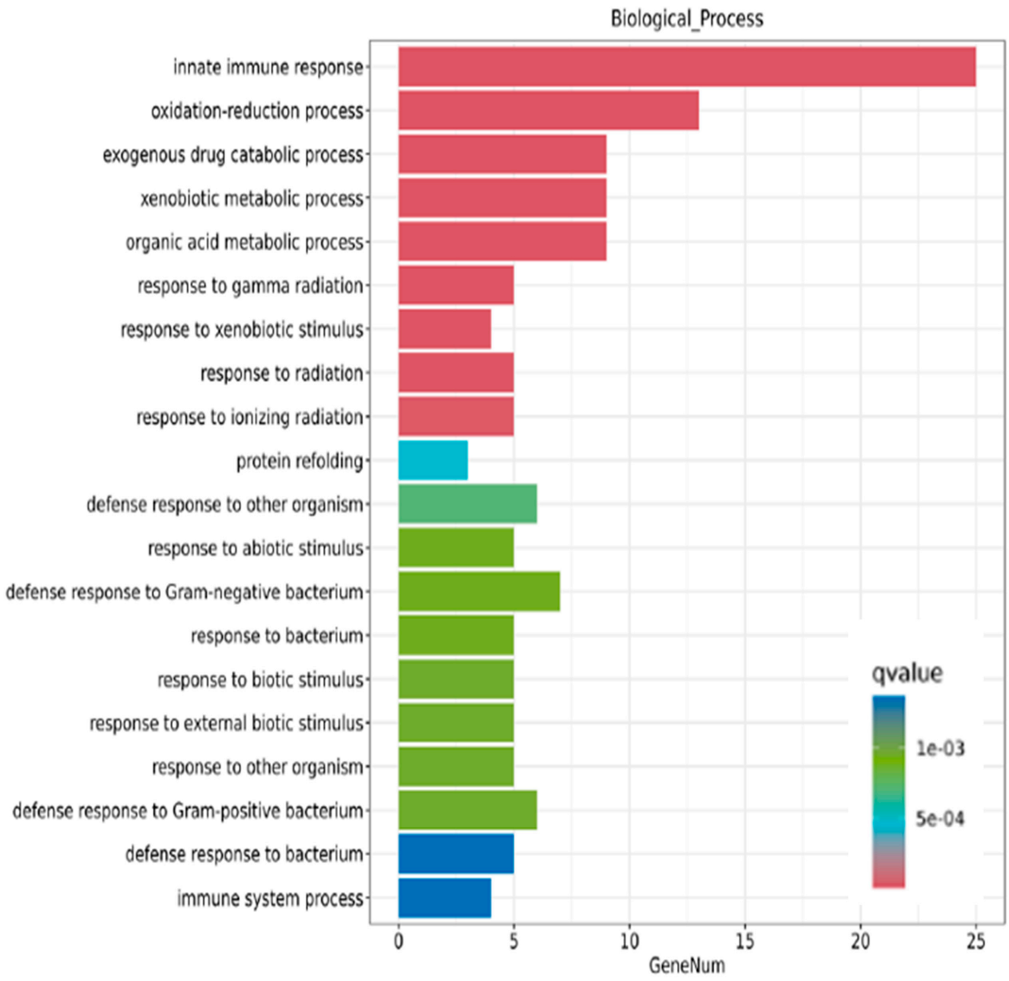

Figure S2. In the biological process, the upregulated genes are mainly enriched in innate immune response and oxidation-reduction process.

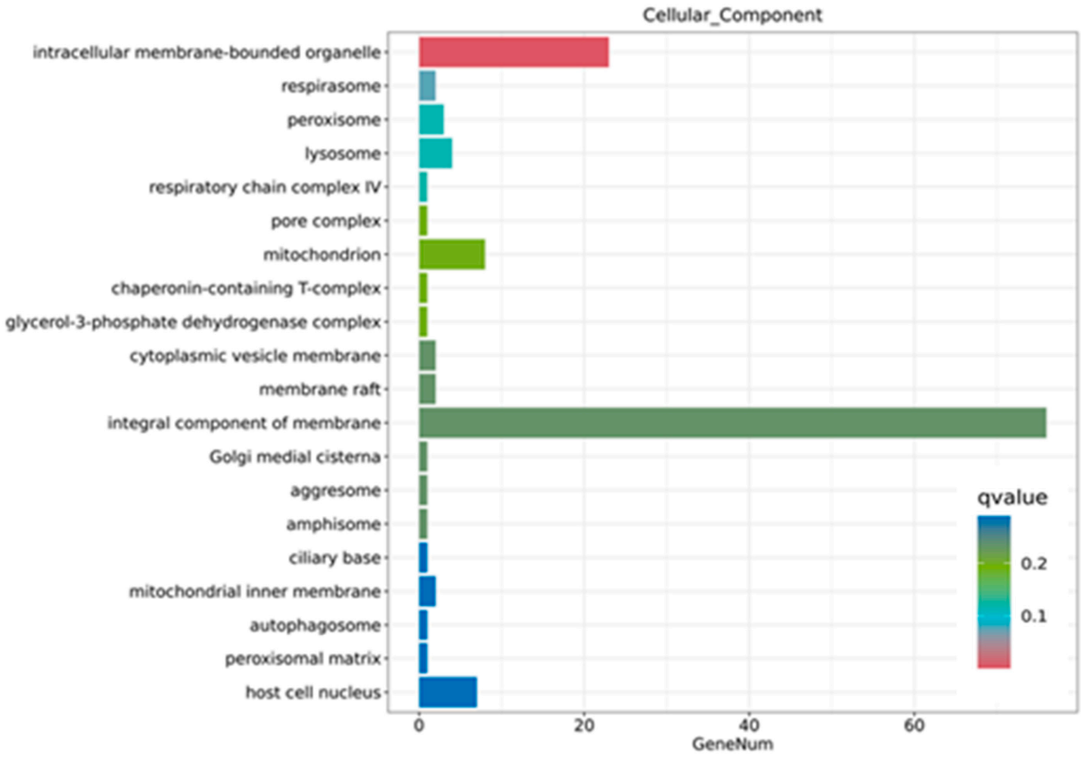

Figure S3. In the cellular component, the upregulated genes are mainly enriched in cytoskeleton and intracellular membrane-bounded organelle.

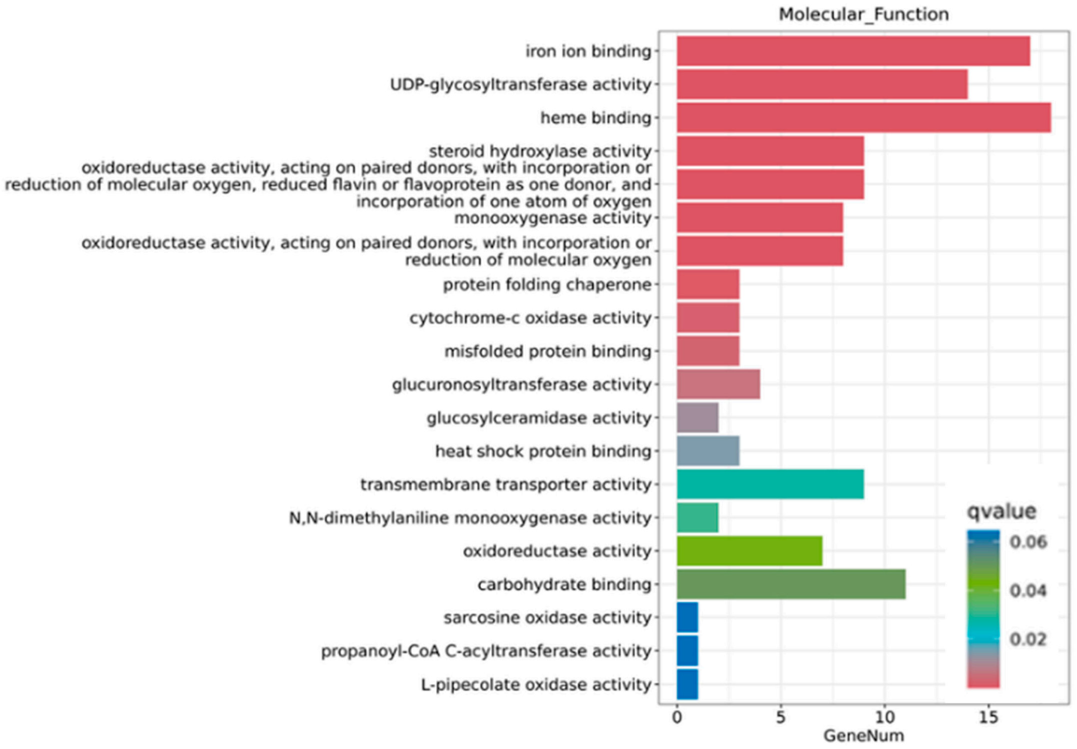

Figure S4. In the molecular function category, the upregulated genes are mainly enriched in heme binding, iron ion binding, and protein serine/threonine kinase activity.

Table S1. The gene sequence for RT-qPCR.

Gene accession number	Gene name	Primer sequence (5' to 3')
**NM_073418.9**	*act-1(F)*	CTACGAACTTCCTGACGGACAAG
	act-1(R)	CCGGCGGACTCCATACC
**NM_001381210.1**	*daf-16(F)*	CTAACTTCAAGCCAATGCCACTA
	daf-16(R)	TCCAGCTTGACTCAGCTCATGTC
**NM_060630.7**	*hsf-1(F)*	TTTGCATTTTCTCGTCTCTGTC
	hsf-1(R)	TCTATTTCCAGCACACCTCGT
**NM_001372824.1**	*skn-1(F)*	AGAGAAATCGACAGTAGCGAGAA
	skn-1(R)	TCGGCTTTTTGAGTTGGATGT
**NM_078363.9**	*sod-3(F)*	AGCATCATGCCACCTACGTGA
	sod-3(R)	CACCACCATTGAATTTCAGCG
**NM_069447.8**	*gst-4(F)*	TCCGTCAATTCACTTCTTCCG
	gst-4(R)	AAGAAATCATCACGGGCTGG
**NM_001392482.1**	*hsp-16.2(F)*	CTGCAGAATCTCTCCATCTGAGTC
	hsp-16.2(R)	AGATTCGAAGCAACTGCACC
**NM_001268666.4**	*fat-6(F)*	GGCAAACCGTGATTTTCACATT
	fat-6(R)	TCACGAGCCCATTCGATGAC
**NM_001029376.6**	*daf-12(F)*	TCCAATGCCAGCTGAAACAACACC
	daf-12(R)	TGGAATGGCTGACACGGTTGAATG
**NM_181901.2**	*nhr-80(F)*	AATTCCGATTTCCAGCTTCTTC
	nhr-80(R)	TCTGCAGATTTGGTGCATACTATAA
**NM_001383536.2**	*fard-1(F)*	CGCATTCGCCAAGAGAAACC
	fard-1(R)	ACGTTGACATTGTCTCGGATGA

Table S2. The 5 genes with the most significant upregulation of gene expression by berberine in *C. elegans*.

Gene name	Log2FC	Gene description *
srz-15	5.55	Predicted to be located in membrane.
cyp-35A1	4.81	Predicted to enable heme binding activity; oxidoreductase activity, acting on paired donors, with incorporation or reduction of molecular oxygen, reduced flavin or flavoprotein as one donor, and incorporation of one atom of oxygen; and steroid hydroxylase activity. Involved in response to xenobiotic stimulus. Predicted to be located in cytoplasm and intracellular membrane-bounded organelle. Human ortholog(s) of this gene implicated in several diseases, including artery disease (multiple); gastrointestinal system cancer (multiple); and lung disease (multiple). Is an ortholog of several human genes including CYP2B6 (cytochrome P450 family 2 subfamily B member 6); CYP2D6 (cytochrome P450 family 2 subfamily D member 6); and CYP2E1 (cytochrome P450 family 2 subfamily E member 1).
irg-6	4.10	Involved in response to gamma radiation.
cyp-35A5	3.22	CYtochrome P450 family; Predicted to enable heme binding activity; oxidoreductase activity, acting on paired donors, with incorporation or reduction of molecular oxygen, reduced flavin or flavoprotein as one donor, and incorporation of one atom of oxygen; and steroid hydroxylase activity. Involved in response to xenobiotic stimulus. Predicted to be located in cytoplasm and intracellular membrane-bounded organelle. Expressed in intestine.
cyp-35B2	3.22	CYtochrome P450 family; Predicted to enable heme binding activity; oxidoreductase activity, acting on paired donors, with incorporation or reduction of molecular oxygen, reduced flavin or flavoprotein as one donor, and incorporation of one atom of oxygen; and steroid hydroxylase activity. Predicted to be involved in organic acid metabolic process and xenobiotic metabolic process. Predicted to be located in cytoplasm and intracellular membrane-bounded organelle.

*Data source: https://wormbase.org/

Table S3. The 5 genes with the most significant downregulation of gene expression by berberine in *C. elegans.*

Gene name	Log2FC	Gene description *
T16G12.10	-2.78	Enriched in MSpppaaa; Z1; Z4; head mesodermal cell; and in male based on RNA-seq and single-cell RNA-seq studies. Is affected by several genes including *mter-4*; *csr-1*; and *rrf-3* based on RNA-seq studies. Is affected by nine chemicals including Alovudine; stavudine; and allantoin based on RNA-seq and microarray studies.
K12H6.9	-2.77	Enriched in ALN; germ line; head neurons; and in male based on proteomic; RNA-seq; single-cell RNA-seq; and microarray studies. Is affected by several genes including *cyc-1*; *ets-4*; and *csr-1* based on microarray and RNA-seq studies. Is affected by eight chemicals including Psoralens; allantoin; and Sirolimus based on RNA-sPredicted to enable hormone activity. Predicted to be involved in signal transduction. Predicted to be located in extracellular region. Expressed in coelomocyte; male gonad; and seminal vesicle.eq and microarray studies.
ins-31	-2.67	Predicted to enable hormone activity. Predicted to be involved in signal transduction. Predicted to be located in extracellular region. Expressed in coelomocyte; male gonad; and seminal vesicle.
K11D12.6	-2.63	Predicted to enable serine-type endopeptidase inhibitor activity.
Y6G8.15	-2.62	Is affected by several genes including *csr-1*; *rrf-3*; and *tph-1* based on RNA-seq studies. Is affected by seven chemicals including Psoralens; allantoin; and Sirolimus based on RNA-seq studies. Is predicted to encode a protein with the following domains: PAN/Apple domain and PAN domain.

*Data source: https://wormbase.org/
